# Cryptococcal Meningitis in an HIV-Negative Puerperal Woman

**DOI:** 10.1155/2021/6665624

**Published:** 2021-05-25

**Authors:** Tomás Robalo Nunes, Helena Pires, Liliana Alves, Ana Guerra, Susana Boavida, Ana Brito, Inês Marques, Nuno Marques

**Affiliations:** ^1^Infectious Diseases Resident, Infectious Diseases Service, Hospital Garcia de Orta EPE, Almada, Portugal; ^2^Consultant in Infectious Diseases, Infectious Diseases Service, Hospital Garcia de Orta EPE, Almada, Portugal; ^3^Consultant in Internal Medicine, Infectious Diseases Service, Hospital Garcia de Orta EPE, Almada, Portugal; ^4^Consultant in Neurology, Neurology Service, Hospital Garcia de Orta EPE, Almada, Portugal; ^5^Head of Department of Infectious Diseases, Infectious Diseases Service, Hospital Garcia de Orta EPE, Almada, Portugal

## Abstract

Cryptococcal meningitis is a common opportunistic infection in HIV-infected patients and other immunocompromised people. Pregnancy, which is a state of relative immunosuppression, can also be a risk factor for the development of cryptococcal meningitis. We report a clinical case of a 41-year-old woman who developed a severe meningeal syndrome after an otherwise normal pregnancy. Cerebrospinal fluid (CSF) cytochemical analysis presented hypoglycorrhachia, high protein levels, and pleocytosis. Cryptococcal antigen tested positive in serum and CSF, and *Cryptococcus neoformans* was identified in the CSF culture. The diagnosis of cryptococcal meningitis was confirmed, and antifungal induction therapy was started with liposomal amphotericin B and flucytosine. After clinical improvement, induction therapy was discontinued, and the patient was discharged under maintenance therapy with fluconazole. While under antifungal maintenance therapy, the patient presented worsening of symptoms and a new brain magnetic resonance showed the development of multiple cryptococcoma. Despite sterile CSF cultures, there was a deterioration of the cytochemical parameters. The diagnosis of immune reconstitution inflammatory syndrome was assumed, and after initiation of corticotherapy, the patient improved considerably. This is a rare case of cryptococcal meningitis in a puerperal woman with a challenging management.

## 1. Introduction

Cryptococcosis is a common opportunistic infection in HIV-infected patients caused by the fungus *Cryptococcus neoformans* and *Cryptococcus gatii* [1]. In fact, extrapulmonary cryptococcosis is an AIDS-defining condition [2].

Other risk factors for the development of cryptococcosis are the chronic use of corticotherapy [1, 3] and other immunosuppressive drugs [1], diabetes [4], cancer and rheumatologic diseases [3, 4], and solid organ transplants, hematologic malignancies, or even chronic renal or lung disease and hepatic failure [3]. In these HIV-negative populations, cryptococcosis mainly affects the lungs and central nervous system (CNS) [3]. Rarely, patients without the traditional immunosuppression risk factors [5], such as pregnant women, can also develop cryptococcosis [6–9].

Clinically, cryptococcal meningitis usually presents as a meningeal syndrome [8], although more indolent when compared with classical bacterial meningitis. In pregnancy, sometimes, an increasing intensity of symptoms after delivery can occur [7], due to immune reconstitution inflammatory syndrome (IRIS) [10].

Cryptococcal meningitis is a therapeutic challenge due to the long duration of antibiotherapy required to eradicate infection, potential significant side effects, low availability of first-line antifungal therapies in many countries, and the uncertain evolution even under therapy [11].

We present a case of cryptococcal meningitis in a puerperal woman with no other known immunosuppression factors. She presented paradoxical clinical worsening under antifungal therapy probably related to IRIS in the postpartum period.

## 2. Case

A 41-year-old Caucasian Portuguese woman, with history of migraine and anxiety, with no chronic medication, was admitted in February 2020 for full-term delivery, which was performed without complications. The patient referred recurrent episodes of headache, photophobia, and nauseas in the two previous months, with resolution using symptomatic medication, as nonsteroidal anti-inflammatory drugs (NSAIDs). She also described having occasional periods of low-grade fever (38°C axillar), with resolution with paracetamol, during the previous month. She denied behavioral changes or focal neurological signs.

After delivery, the patient developed a new episode of headache with similar characteristics as described before but with increased intensity. She had a tympanic temperature of 37.7°C and Glasgow Coma Scale (GCS) 15, although the anamnesis was affected by the intense pain described and aggressive behavior. She showed no abnormalities in cranial nerve evaluation, motor and sensory system, deep tendon reflexes, and cerebellum functions. She presented nuchal rigidity with a positive Brudzinski sign. Head computed tomography (CT) showed no acute or chronic abnormalities. Lumbar puncture revealed high CSF opening pressure (37 cm H₂O) and xanthochromia. The CSF was cloudy, with raised protein levels (284 mg/dL), pleocytosis with mononuclear predominance (556 cells/*µ*L), and hypoglycorrhachia (<2.0 mg/dL glucose). Direct microscopy examination of the CSF was suggestive of *Cryptococcus* spp. infection, and the CSF cryptococcal antigen was positive (1/40960). The serum cryptococcal antigen was also positive (1/12560). An HIV 4^th^ generation test performed during the third trimester and on admission was negative; CD4 lymphocyte counts, complement, and immunoglobulins were within normal range.

**Figure 1:**
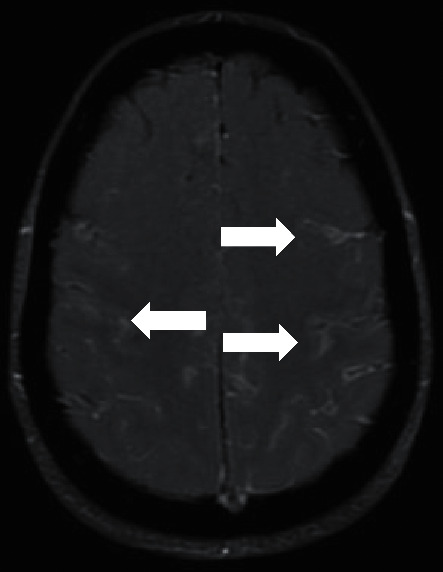
Brain MRI: T1, after gadolinium administration; arrows: leptomeningeal enhancement.

The diagnosis of cryptococcal meningitis was confirmed, and antibiotherapy was started with liposomal amphotericin B 250 mg/daily and flucytosine 1500 mg, 4 times/day, weight-adjusted (65 kg). Additionally, she was kept under empirical antibiotherapy for community-acquired acute meningitis with ceftriaxone and ampicillin for 3 days, which was stopped after the CFS bacteriological cultures became negative. The fungal CFS cultures were positive for *Cryptococcus neoformans*, with negative blood cultures. Brain magnetic resonance imaging (MRI) showed signs of extensive meningitis (Figure 1), with a parenchymal image of small dimensions, with poorly defined characteristics on the right occipital region. The chest X-ray was normal. The patient started bromocriptine as she would not be allowed to breastfeed during the expected long antibiotherapy course.

Although several lumbar punctures were performed, the CSF opening pressure continued to be extremely high (maximum >50 cm H₂O), and optical nerve edema was observed in optical coherence tomography. Considering this presentation, a communicating hydrocephalus secondary to the cryptococcal meningitis was diagnosed, and a ventriculoperitoneal shunt was placed on the 9^th^ day of antifungal therapy. Even after this procedure, the pain management was challenging, with the need for repeated CSF evacuation and opioid medication.

After approximately 5 weeks of induction therapy, with three consecutive negative CSF fungal cultures and with symptomatic and CFS cytochemical characteristics improvement, the ventriculoperitoneal shunt was disconnected. Antifungal therapy was changed to a maintenance scheme with oral fluconazole 800 mg/day, and the patient was discharged clinically stable, apyretic, and without neurological symptoms.

**Figure 2:**
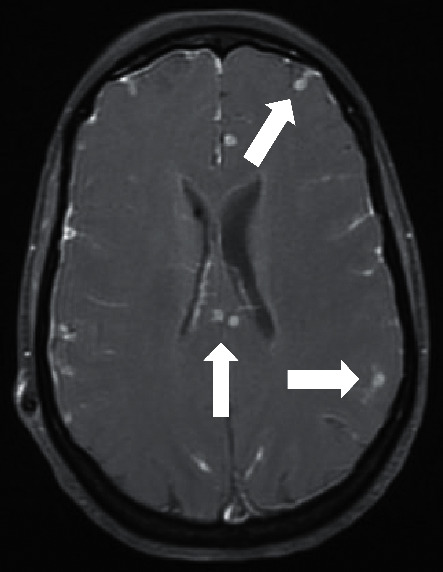
Brain MRI: T1, after gadolinium administration; arrows: multiple cryptococcoma.

After 2 months of maintenance therapy with fluconazole, the patient was readmitted due to worsening of headache, photophobia, diplopia, and confusion. She was prostrated, with GCS 14, disoriented in time and space, with dysarthria, convergent strabismus, nystagmus, and peripheric facial paralysis. The head CT scan showed signs of worsening enlargement of the cerebral ventricles. A lumbar puncture confirmed high CSF opening pressure, with worsening of the protein levels (462 mg/dL), hypoglycorrhachia (36 mg/dL), and pleocytosis (117 cells/*µ*L) with mononuclear predominance. To control the high intracranial pressure, the ventriculoperitoneal shunt was reopened. Liposomal amphotericin B and flucytosine were restarted, assuming possible recurrent cryptococcal infection, and she was treated empirically with meropenem and vancomycin for possible nosocomial meningitis. Brain MRI showed worsening of the meningitis signs and several new intraparenchymal cryptococcoma, with no encephalic herniation (Figure 2).

As the CFS and blood cultures were persistently negative and the patient was previously relatively immunosuppressed in the context of pregnancy, the diagnosis of immune response inflammatory syndrome (IRIS) was considered, which would explain the paradoxical clinical worsening under antifungal therapy. For this reason, corticotherapy was also started.

After six weeks of therapy with liposomal amphotericin B, flucytosine, and corticotherapy (tapering scheme), the patient improved considerably, revealing no neurological deficits. A new encephalic MRI showed that the cryptococcoma were in resolution phase with reduced size.

For this reason, the described therapeutic scheme was discontinued, and maintenance therapy with oral fluconazole was started. After more than 6 months under this therapy, the patient is asymptomatic and radiologically stable. There was no evidence of infection in the newborn.

## 3. Discussion

Cryptococcosis is an opportunistic infection that commonly affects immunocompromised patients [1]. HIV-infected people are the most important group with population studies showing that around 89% of cryptococcosis cases in some states of the United States of America [4] and 85% in Brazil occurred within this population [1].

However, even immunocompetent patients can be affected by CNS cryptococcosis, with high levels of mortality [5]. Pregnant women have been one group of patients affected by cryptococcosis, not only in CNS [8, 9, 12–14] but also as cryptococcal pneumonia [15] or osteomyelitis [16].

The regulation of the immune system during pregnancy is extraordinarily complex. More than a simple state of immunosuppression, there is a complex modulation of the immune system that implies different responses to the invasive pathogens, according to the stage of pregnancy [17]. However, in general, it is stated that, during pregnancy, a relative level of immunosuppression occurs in order to prevent fetal rejection [8, 10, 15], in response to the paternally derived histocompatibility antigens [8]. In this context, some natural anti-inflammatory responses are increased, with downregulation of Th-1 cytokines (eg., INF-*γ*) [10]. Moreover, maternal hormones, such as progesterone, can also modulate the immune system during pregnancy [10]. In fact, progesterone is seen as one of the most important hormones regulating the inflammatory response during pregnancy. Its anti-inflammatory effects improve the uterine environment for the fetus implantation and reduce different inflammatory pathways in the different pregnancy stages [18]. This anti-inflammatory context can increase the risk of different infections. Specifically, one important predisposing factor for the development of cryptococcosis in pregnant woman is the changing of maternal T cells and other immune cells' activity [8, 15].

The diagnosis of cryptococcal disease is a challenge, particularly during pregnancy, due to insidious symptoms, with low-grade fever and intermittent headaches [7]. Our patient, in the two previous months before delivery, described similar symptoms. Moreover, it was only immediately after birth that the full symptomatic intense headache with behavioral changes ensued. One possible reason is the high level of endogenous production of glucocorticoids in the third trimester of pregnancy, which may mask the symptoms; after delivery, when glucocorticoid levels become normalized [7] and other anti-inflammatory responses reverted [10], the most intense symptoms develop.

Treatment recommendations are mostly based on HIV-infected patient studies and depend on clinical and radiological evolution. For non-HIV-infected, nontransplant patients, the Infectious Diseases Society of America recommends a minimum of four weeks of induction therapy with liposomal amphotericin B and flucytosine in cases of cryptococcal meningitis and at least six weeks in patients with cryptococcoma [19]. The American Thoracic Society recommends treatment during two weeks for patients with CNS involvement [20]. However, in developing countries where these antifungal therapies are not available, high-dose monotherapy fluconazole is also an option to be considered [11], probably due to its good penetration in the brain parenchyma [21]. In addition, it has also been reported that a woman diagnosed with cryptococcoma in the immediate postpartum period, with intolerance to amphotericin (body rash and severe hypokalemia and hypomagnesemia with ventricular arrhythmia), was also successfully treated with high-dose fluconazole monotherapy [12]. Fortunately, pregnant women who are correctly diagnosed and treated usually have a favorable prognosis, with no consequences to the fetus [8, 14]. Transplacental transmission of *Cryptococcus* is rare [9], although it has been described, mostly in HIV-infected pregnant women [22].

Our patient presented symptomatic relapse and development of multiple cryptococcoma even under maintenance therapy. These could be secondary to persistent infection due to fluconazole resistance, nonadherence to therapy, or IRIS [11].

In this case, *Cryptococcus neoformans* was susceptible to amphotericin B, and the minimal inhibitory concentration (MIC) was 8 *μ*g/mL for fluconazole. Although there are no defined breakpoints for *Cryptococcus* and fluconazole, clinical studies suggest that isolates with MIC ≤8 *μ*g/mL can be considered susceptible, with dose-dependent sensitivity with MICs between 16–32 *μ*g/mL and resistant when MIC ≥64 *μ*g/mL [11, 23]. Furthermore, according to Aller et al., in general, response to fluconazole maintenance therapy can be expected to be better when MIC is ≤16 *μ*g/mL [24].

Although the distinction between treatment failure and IRIS is not always clear [11], IRIS is a well-described phenomenon in the postpartum period [10]. In fact, some autoimmune disorders, such as rheumatoid arthritis and Graves' disease, tend to undergo remission during pregnancy and aggravate in the postpartum period [10]. Tuberculosis, cryptococcosis, hepatitis B, and herpes virus infection may get worse or have flares in the postpartum period [10]. In our patient, the finding of persistent negative CSF cultures during clinical worsening, with high CSF blood cell counts, and the remarkable response to corticotherapy were highly suggestive of IRIS [11].

After more than four months under maintenance therapy with fluconazole and after stopping the corticotherapy, the patient is clinically and radiologically stable. This represents an uncommon and challenging clinical case.

## Data Availability

All references used to support our conclusions can be obtained from usual medical databases, such as PUBMED.

## Additional Points

*Relevance*. The authors believe this case report is relevant for all clinicians, not only committed with Infectious Diseases but also Internal Medicine and Obstetrics, due to the rarity of the infectious agent in pregnant women without other risk factors and to the challenging diagnostic and therapeutic approach.
